# Immunologic Memory in Pregnancy: Focusing on Memory Regulatory T Cells

**DOI:** 10.7150/ijbs.70629

**Published:** 2022-03-06

**Authors:** Yu-Jing Zhang, Li Shen, Tao Zhang, Kahindo P. Muyayalo, Jing Luo, Gil Mor, Ai-Hua Liao

**Affiliations:** 1 Institute of Reproductive Health, Center for Reproductive Medicine, Tongji Medical College, Huazhong University of Science and Technology, Wuhan, P.R. China.; 2 Department of Obstetrics and Gynecology, Liyuan Hospital, Tongji Medical College, Huazhong University of Science and Technology, Wuhan, P.R. China.; 3 Department of Obstetrics and Gynaecology, Faculty of Medicine, The Chinese University of Hong Kong, Prince of Wales Hospital, Shatin, Hong Kong, P.R. China.; 4 C.S. Mott Center for Human Growth and Development, School of Medicine, Wayne State University, Detroit, MI, USA.

**Keywords:** regulatory T cells, memory, pregnancy, preeclampsia, gestational diabetes mellitus

## Abstract

A successful pregnancy requires the maternal immune system to tolerate an allogeneic fetus. The incidence of preeclampsia and other complications related to impaired fetal tolerance is lower during the second pregnancy than during the first pregnancy. At the same time, compared with normal pregnant women in the previous pregnancy, patients with pregnancy complications in the previous pregnancy also have an increased risk of the disease when they become pregnant again. This difference may be related to the immunological memory of pregnancy. Regulatory T cells (Tregs) are immunosuppressive CD4^+^ T cells that play a predominant role in maintaining immune tolerance. In addition, Tregs possess immunological memory properties, including fetal or paternal-specific memory Tregs and Tregs expressing memory cell makers, forming an immunoregulatory memory against fetal antigens. In this review, we provide an overview of the characteristics of memory Tregs in pregnancy, evidence regarding the existence of memory Tregs in human pregnancy, as well as in mouse models. We also discuss the mechanism of memory Tregs induction, maintenance, and action. In addition, we described their changes during the first pregnancy, second pregnancy, postpartum, and pathological pregnancy in order to provide new targets for the diagnosis and treatment of pregnancy related diseases.

## Introduction

"Immune memory" implies that during the process of immunity, following reaction with a specific antigen, contact, and stimulation by the same antigen can quickly initiate secondary immunity to induce a stronger immune response. The pregnancy outcomes of secondary pregnancies are more favorable than that of primary pregnancies. The incidences of fetal growth restriction, fetal death, and low birth weight were lower in subsequent pregnancies 1, 2. Simultaneously, the incidence of preeclampsia (PE) and other complications associated with impaired fetal tolerance in multiparas were lower than those in nulliparas 3, 4. Multiparas are usually defined as women who have given birth at least twice to an infant, and nulliparas are defined as women who have never given birth. However, a second pregnancy with a different partner has an incidence similar to that of the first pregnancy 5, 6. This might be due to immune memory during pregnancy. Thus, multiparous women may "remember" the antigen of the previous pregnancy and rapidly produce "protective" reactions such as tolerance when they are pregnant again to achieve a more favorable pregnancy outcome.

With the increase of the number of pregnancy losses, the incidence of re-abortion increases. Compared with normal pregnant women in the previous pregnancy, patients with pregnancy complications in the previous pregnancy also have an increased risk of the disease when they become pregnant again. This further shows that good pregnancy memory may lead to a good pregnancy outcome, while bad pregnancy memory may lead to adverse pregnancy outcomes. Recent studies have revealed that some immune memory cells are present in the decidua and peripheral blood during pregnancy, including B cells 7-9, T cells 1, 10-12, and natural killer (NK) cells 13, 14. These cells expand rapidly after pregnancy, especially after the second pregnancy, and play different roles during pregnancy.

Regulatory T cells (Tregs) are immunosuppressive CD4^+^ T cells that play a pivotal role in maintaining self-tolerance by preventing immune responses against autoantigens 15, 16. In mice, the number of Tregs selectively accumulates after pregnancy and remains at a high level for a long time after delivery, forming an immunomodulatory memory for fetal antigens. Moreover, the accelerated expansion of Tregs during the second pregnancy was almost entirely driven by the proliferation of fetal-specific forkhead box P3^+^ (Foxp3^+^) cells retained in the previous pregnancy. These fetal-specific Tregs can detect functional loss, which may be beneficial for maintaining pregnancy immune tolerance 17. In humans, memory Tregs have also been found during early pregnancy 18. Therefore, the importance of memory Tregs during pregnancy has been widely concerned and worthy of discussion.

The present review aimed to furnish novel research directions and possible therapeutic targets to further investigate memory Tregs in normal pregnancy and pregnancy-related complications. Herein, we summarized evidence of memory Tregs in humans and mouse models during pregnancy, the generation and maintenance of memory Tregs during pregnancy, and the role of memory Tregs in normal and pathological pregnancies.

## Overview of Tregs in pregnancy

A successful pregnancy requires the maternal immune system to tolerate an allogeneic fetus 19, 20. Disorders impacting immune tolerance may result in pregnancy loss, fetal growth restriction, premature birth, and PE 21-23. Present in both the basal decidua and parietal decidua at the maternal-fetal interface 24, 25, Tregs cells are involved in the regulation of autoimmunity and tolerance toward the fetus by suppressing maternal immune responses 26-29. The expansion of Tregs starts extremely early during pregnancy 30. After this early increase, decidual Tregs remained elevated in the second trimester and then reduced before birth 31, 32.

In mice, the absence of Tregs in the first trimester of pregnancy can lead to pregnancy failure due to fetal immune rejection, either antibody-related Tregs depletion or CD25 depletion 33, 34. Indeed, in the abortion-prone model of CBA/J × DBA/2 35, it was observed that the embryo resorption rate increased, along with the simultaneous exhaustion of Tregs 10. The adoptive transfer of Tregs from normal pregnant mice can prevent fetal rejection 36 and significantly reduce the fetal absorption rate 37, 38. In addition to immune tolerance during the first trimester, depletion of functional Tregs in the third trimester can lead to premature delivery and adverse neonatal outcomes, which can be rescued by the adoptive transfer of Tregs 39.

In humans, the same phenomenon has been observed. A high proportion of Tregs cells is present in the peripheral blood and decidua of early pregnancy 40, expressing CD152 (cytotoxic T-lymphocyte-associated protein-4, CTLA-4). This increased Tregs cells number plays an important role in immune tolerance by effectively inhibiting the proliferation of autologous T cells 41. In contrast, a lack of Tregs can result in pregnancy failure. The proportion of CD4^+^CD25^bright^ T cells in the decidua of women with spontaneous abortion was significantly lower than that in women with induced abortion 41. The frequency of Foxp3^+^CD4^+^ T cells in miscarriage with a normal embryo was significantly lower than those in miscarriage with an abnormal embryo 42. In normal fertile non-pregnant women, peripheral Tregs expand in the late follicular phase, followed by a dramatic decrease in the luteal phase. However, the number of Tregs in women with recurrent spontaneous abortion was similarly low during follicular and luteal phases. In addition, their function was reportedly reduced 43. Through the expression of protective factors including interleukin (IL) -10, transforming growth factor-β (TGF-β), heme oxygenase-1, indoleamine 2,3-dioxygenase, leukemia inhibitory factor, and negative costimulatory molecule (like CTLA-4, TIGIT) 44, Tregs at the maternal-fetal interface create a tolerant microenvironment and play an important role in avoiding fetal allograft rejection.

## Memory Tregs in mice models

Appropriate animal models help explore the mechanisms of Tregs during pregnancy memory. Rosenblum et al. 45 first found Treg memory in transgenic mice. They crossed transgenic mice expressing a membrane-bound form of Ovalbumin (Ova) under the control of a tetracycline response element to transgenic mice expressing the tetracycline transactivator protein under the control of the keratin 5 promoters. Then the model antigen, Ova, could be inducibely expressed within the skin. It was further found that exposure to tissue autoantigen could lead to the activation of autoreactive Treg cells produced by autoantigen expression in the thymus. Activated Treg cells persist in target tissues and inhibit autoimmune response when repeatedly or chronically exposed to tissue autoantigens. In mice, the key challenge to examine Tregs in pregnancy memory is distinguishing between fetal and paternal-specific Tregs. I-A^b^2W1S_55-68_ peptide and β-actin were co-expressed in transgenic male mice and then conceived with non-2W1S-expressing B6 females. The I-A^b^2W1S_55-68_ peptide can replace fetal antigens. Endogenous maternal Tregs that replaced fetal antigens could be identified by major histocompatibility complex (MHC) class II tetramer enrichment. 2W1S^+^ Tregs are considered fetal-specific Tregs 17.

Another method to identify paternal antigen-specific Tregs is to use the MLS-1^a^ superantigen for Treg expression on DBA/2 cells and recognition by the T cell receptor Vβ6. Tregs expressing Vβ6 are considered MLS-1^a^ -specific Tregs 46. These are called PA-specific Tregs (CD4^+^Foxp3^+^Vβ6^+^), which are considered paternal antigen-specific Tregs 47. Although the second method has not been used to evaluate pregnancy memory, using this method, the authors put forward unique views regarding the effect of semen exposure on paternal alloantigen reactivity.

In diphtheria toxin receptor (DTR) mice, the target cells express DTR through gene editing and are conditionally eliminated by injecting diphtheria toxin (DT). In 2012, Rowe et al. 17 first applied this model to evaluate fetal-specific memory Tregs during pregnancy in mice. Foxp3^DTR/DTR^ transgenic mice were first constructed, and then Foxp3^+^-specific ablation mice were obtained by DT injection. The authors also adoptively transferred CD45.1CD4 cells from Foxp3^DTR/DTR^ mice donors to CD45.2CD4 receptor mice. Next, CD45.1Foxp3^+^CD4 cells in mice were specifically eliminated postpartum to assess the critical role of fetal-specific Tregs produced during pregnancy. This mouse model was also used in a subsequent study of Tregs in idiopathic preterm birth. Interestingly, the authors established a new mouse model by intraperitoneally injecting Foxp3^DTR^ at 14.5 days postcoital (dpc) with 25 or 50 μg/kg DT, followed by 5 or 50 μg/kg/day DT 24 h later until delivery. The objective was to create a partial or total Foxp3 depletion model during pregnancy, where partial Foxp3 depletion during the second trimester approximated premature delivery in mice 39. This provides critical insight. As DT depletion is transient, a mouse model with partial or total Foxp3 depletion, induced by injecting DT at different doses, may be more conducive for assessing the effects of Tregs at different pregnancy stages, as well as for constructing a more appropriate animal model. Furthermore, it may help investigate the role of memory Tregs during different stages of pregnancy.

Given ethical restrictions during human pregnancy assessments, current research remains focused on elucidating changes and functions of memory Tregs in peripheral blood during different pregnancy periods. Specific markers for fetal-specific Tregs are scarce. In addition, reports on altered Treg levels in the decidua during different gestational periods are lacking.

## Characteristics of memory Tregs in pregnancy

Based on the literature, memory Tregs in pregnancy are mainly classified by two strategies. One is to classify memory Tregs by adding special memory markers to Tregs. This classification is predominantly based on the experience of memory T cells. The second strategy involves the inclusion of fetal or paternal-specific Tregs, which reportedly play an extremely important role in immune tolerance and memory of pregnancy.

Naïve T cells express CD45RA^+^, and memory T cells express CD45RO^+^ 48. Memory T cells can rapidly differentiate into effector cells and new memory T cells after antigenic stimulation 49. According to their locations and functions, they can be divided into central memory T cells (T_CM_), effector memory T cells (T_EM_), and tissue-resident memory T cells (T_RM_). T_CM_ expresses chemokine receptor (CCR) 7^+^ in secondary lymphoid organs, but not in non-lymphoid tissues 50 such as lymph nodes and spleen. The other two memory T cells are CCR7^-^ and are expressed in non-lymphoid tissues such as the skin, lung, or intestine 51. After T_CM_ activation, CD69 (a marker of activated T cells) is expressed on the cell surface, and T_CM_ cells irreversibly lose CCR7 expression and proliferate to T_EM_ 48. Therefore, CCR7 is typically employed to distinguish between central and effector subtypes of memory T cells.

Recent studies have confirmed the presence of Treg_EM_ (CD25^+^CD45RA^-^CD62L^-^) and Treg_CM_ (CD25^+^CD45RA^-^CD62L^+^) in peripheral blood 52, 53, with significant differences in functions. The frequency and function of Treg_EM_ are reportedly suppressed in patients with delayed fracture union when compared with those of Treg_EM_ in normal patients. Treg_EM_ expressed more TGF-β and IL-10 when stimulated, which may be beneficial for the occurrence of anti-inflammatory effects 52. However, evidence indicating the presence of Treg_EM_ and Treg_CM_ in human pregnancy is lacking, further studies are needed to show the existence, changes, and function of Treg_EM_ and Treg_CM_ in human pregnancy.

In mice, when the immune system recognizes tumor cells, CD44hi memory Tregs rapidly establish immune tolerance. The relative speed of these Tregs and effector T cells (Teffs) reaction determines the result of the antitumor immune response: tolerance or rejection 54. Chen et al. 10 defined CD4^+^Foxp3^+^CD44^high^CD62L^low^ as activated/memory Tregs (amTregs) in the draining lymph node (dLN) during pregnancy. AmTregs were found to be present in both lymph nodes draining deep tissues and peripheral blood in non-pregnant mice 55, 56. These cells can recognize autoantigens and avoid the development of autoimmune diseases 57. Fetal and tumor microenvironments have similar immune tolerance mechanisms. Through the overall comparison of fetal and tumor microenvironments by mouse transcriptome, it is revealed strikingly similar in the expression of numerous immune-related pathways, including dynamic upregulation of Treg-related pathways in the pregnant uterus^58^. In addition, amTregs have the advantage of regulating the immune response of antitumor and anti-fetal effects, which is beneficial for embryo implantation and inducing immune tolerance during pregnancy^10^, ^54^.

In human pregnancies, studies included memory Tregs (CD45RA^-^CD45RO^+^) and memory Treg subsets differentiated by human leukocyte antigen (HLA)-DR^+/-^ and CD31^+/-^ 18, 59-61. HLA-DR is a type of HLA class II molecule, the others being HLA-DP and HLA-DQ and they are expressed on many antigens presenting cells, including monocytes, dendritic cells, and B cells. HLA-DR plays a role in antigen presentation 62. And HLA-DR^+^ is typically employed as an activation marker for T cells and Tregs 63. HLA-DR^+^ Tregs were found to express higher levels of Foxp3 and induce stronger inhibition than Tregs with HLA-DR^-^ 64. Schober et al. 59 divided the total CD4^+^CD127^low+/-^CD25^+^ Foxp3^+^ Tregs pool into four different Treg subgroups, including naïve Tregs (nTregs, CD45RA^+^ Tregs), DR^-^Tregs (HLA-DR^-^CD45RA^-^ memory Tregs), DR^low+^ Tregs (HLA-DR^low +^CD45RA^-^ memory Tregs), and DR^high+^ Tregs (HLA-DR^high+^CD45RA^-^ memory Tregs).

CD31 was also used during human pregnancy to define memory Treg subsets, including CD31^+^ and CD31^-^ memory Tregs 60. CD31 is a platelet endothelial cell adhesion molecule-1, expressed on the surface of human granulocytes, monocytes, and platelets. Moreover, this molecule is enriched at the junction of the endothelial cells 65. CD31 may be involved in leukocyte migration, angiogenesis, and integrin activation. In addition, its interaction with a heterophilic counter receptor on T cells can interfere with T cell activation and inhibit the response of T cells 66.

During pregnancy, some fetal or paternal-specific Tregs have been shown to possess memory. As early as 2008, Tilburgs et al. 67 reported that compared with peripheral blood, CD4^+^CD25^bright^ T cells in full-term basal decidua and parietal decidua significantly enhanced the inhibition of fetal-specific umbilical cord blood cells. However, no difference was observed in the inhibition of third-party umbilical cord blood cells. The authors suggested that CD4^+^CD25^bright^ T cells in maternal peripheral blood had specific "memory" to fetal-specific umbilical cord blood cells. In 2012, Rowe et al. 17 reported, for the first time, that repeated pregnancy initiated the accelerated accumulation of maternal Foxp3^+^ cells in pregnant mice. These cells were derived from pre-existing fetal-specific maternal Tregs retained from previous pregnancies, persisting post-delivery and maintaining the protective and regulatory memory of fetal antigens. Moreover, CD44 (expressed on activated and memory T cells) was increased in fetal-specific CD4^+^ T cells and maintained a 10-fold increase when compared with the non-pregnant control for 100 days after delivery. Subsequently, Gomez-Lopez et al. 39 determined the role of Tregs in preterm birth and suggested that complete depletion of Tregs in the second pregnancy had a more deleterious effect on neonatal survival than depletion during the first pregnancy. The authors concluded that pregnancy could imprint protective and regulatory memory in Tregs.

At present, in mice and humans, the reported memory Tregs during pregnancy include fetal-specific Tregs, amTregs, DR^-^Tregs, DR^+^Tregs, DR^low+^ Tregs, DR^high+^ Tregs, CD31^+^ memory Tregs, CD31^-^ memory Tregs. Table 1 summarizes the research on memory Tregs during pregnancy. It includes the grouping methods of memory Tregs, surface markers, material location, the proportion of different memory Tregs in healthy women, and the main conclusions of the study.

## Memory Tregs in normal pregnancy

The changes in immune memory cells at different stages of pregnancy might help elucidate the function of immune memory cells during different stages of pregnancy. In mice, Rowe et al. 17 revealed that in spleen and lymph node cells, the fetal-specific Tregs increased with progressing pregnancy and peaked at 48 h postpartum, attaining levels 100 times higher than those observed before pregnancy; this was followed by a gradual decrease slowly that could be detected until day 100 post-delivery. Moreover, 2W1S^+^Foxp3^+^ cells (considered to be fetal-specific Tregs) upregulated Ki67, a proliferation marker during pregnancy. Although Ki67 expression decreased post-delivery, the proportion of 2W1S^+^Foxp3^+^ cells on day 100 post-delivery remained twice that observed before pregnancy. This finding may be related to the mechanism via which fetal-specific maternal Tregs proliferate during pregnancy and persist after delivery 17. In the uterus of allogenic female BALB/c, the number and frequency of amTregs from day post-implantation (dpi) 6 to dpi 10 continued to increase in dLN, but not in non-dLN, when compared with the non-pregnant virgin controls 10.

In humans, HLA-DR^+^ memory Tregs exhibited significantly decreased inhibitory activity in the peripheral blood of normal pregnant women when compared with that in non-pregnant women. However, no significant differences were observed in HLA-DR^-^ memory Tregs 18. Treg subsets in the peripheral blood of five women with successful *in vitro* fertilization (IVF)/intracytoplasmic sperm injection (ICSI) pregnancy were analyzed from the beginning of pregnancy, revealing that nTregs transformed into DR^+^ and DR^-^ memory Tregs. The transformation peaked at approximately seven weeks of pregnancy and then reversed until term 18. During the early stages of pregnancy, trophoblast invasion and blood vessel formation mainly occur at the maternal-fetal interface, while Tregs inhibit inflammation and support maternal vascular adaptation to promote trophoblast invasion and placenta into the maternal blood supply 32. Thus, the increase in memory Tregs during early pregnancy may play a crucial role in early embryo implantation and development. The study by Schlossberger et al. 18 also discovered an interesting phenomenon, in which the total Tregs were not altered with age, but subsets of DR^+^ and DR^-^ memory Tregs increased with age. As these changes occurred simultaneously with age-related fertility decline, it appears that in addition to ovarian aging, age-related changes in the Treg pool composition might be related to female fertility loss. In addition, compared with non-pregnant women, there was a decrease in the percentage of DR^high+^ CD45RA^-^ and DR^low+^CD45RA^-^ Tregs and an increase in the percentage of naïve DR^-^CD45RA^+^ Tregs during normal pregnancy from 10 to 20 weeks. These Treg subgroups remained stable until the term. Among them, DR^high+^CD45RA^-^ Tregs showed stronger inhibitory activity 68. In addition, this phenomenon is reversed at the initiation of natural-term delivery 69.

In a study assessing CD31^+^ memory Tregs, at the beginning of pregnancy, the proportion of recent thymic emigrant-regulatory T cells (RTE-Tregs, CD45RA^+^CD31^+^ Tregs) and CD31^+^ memory Tregs (CD45RA^-^CD31^+^ Tregs) decreased significantly, while the proportion of mature nTregs (CD45RA^+^CD31^-^ Tregs) remained unaltered. Furthermore, the complementarity of CD45RA^-^CD31^-^memory Tregs increased 60. CD31^+^ is mainly expressed in CD45RA^+^ T cells, which interferes with T cell activation. This may underlie the decreased proportion of CD31^+^ memory Tregs during pregnancy. Compared with non-pregnant women, CD4^+^CD45RA^-^Foxp3^++^ Tregs and CD4^+^CD45RA^+^Foxp3^+^ Tregs decreased during pregnancy (20w and 32w) and increased on day 4 post-delivery; however, the level remained lower than that in non-pregnant women 61.

In conclusion, during the early pregnancy, CD31^+^ memory Tregs decreased, but the DR^+^ Tregs, DR^-^ Tregs, CD31^-^ memory Tregs in humans and amTregs, fetal-specific Tregs in mice increased. During the second and third trimesters of pregnancy, fetal-specific Tregs in mice increased, and DR^+^ Tregs decreased in humans. The changes in memory Tregs during and after pregnancy in mice and humans are shown in Table 2.

## Generation and maintenance of memory Tregs during pregnancy

All lymphocytes, especially those in the T cell lineage, have a sequence from naïve to effector to memory 70. Different T cell subsets are activated through distinct pathways. CD8^+^ T cells can persist as self-renewal and numerically stable cell populations, meeting the most stringent definition of "memory." In contrast, the maintenance of CD4^+^ T cells is considered unstable and usually requires sustained low-level antigen stimulation 71, 72.

Thornton et al. 73 demonstrated that Helios is expressed in all thymocytes in the double negative stage 2 of thymic development, and may provide a basis for the source of Tregs. It has been reported that the expression of Helios (also known as IKZF2) in maternal Tregs with 2W1S fetal-specificity was gradually downregulated, decreased to as low as 40% during the third trimester of pregnancy, and then slowly increased post-delivery 17. Moreover, it provides potential perspectives for the source of fetal-specific Tregs 17.

A recent mouse study has shown that Foxp3^+^ Tregs specific for paternal antigens were produced outside the thymus and accumulated in the placenta. The study reported that female mice with impaired extrathymic Treg induction exhibited increased fetal absorption 74. Peripheral-derived Tregs (pTregs) are observed by Samstein et al. 74 from day E12 of gestation in mice. They proved that the lack of pTreg cells will lead to the increase of spontaneous abortion. Tilburgs et al. 67 suggested that fetal-specific Tregs were preferentially recruited from maternal peripheral blood to the maternal-fetal interface, where they may contribute to the local regulation of fetal-specific responses and further contribute to pregnancy outcomes. Therefore, fetal-specific Tregs in mice might be generated outside the thymus, but more conclusive evidence still needs to be accumulated.

To date, factors known to induce memory Tregs during pregnancy include paternal semen induction, fetal cell induction, fetal microchimerism induction, and cytokine production.

### Paternal semen

Before pregnancy, female reproductive tissues, especially immune cells, first come into contact with male semen. Paternal antigens may be recognized by female immune cells during mating and can further produce memory.

Using a mouse tumor inoculation model and delayed-type hypersensitivity in mice, Robertson et al. 75 reported that sperm in seminal plasma and semen played an important role in inducing paternal antigen-specific tolerance. Accordingly, exposure to semen during mating can promote functional tolerance to paternal alloantigen. This effect may be mediated by the expansion of the Treg pool. The size of peptides presents in DBA/2 < 10 kD boosted the abortion rate. These discuss the role of seminal plasma peptides on the establishment of immune tolerance to the fetus 76. Another study showed that, after mating with 2W1S^+^ male mice, the number of 2W1S^+^ Tregs was increased in infertile female mice (infertile female mice were constructed by irradiation with 100 rads); however, these levels were lower than those observed in pregnant mice 17. This provides a new concept for the formation of semen-induced Treg immune memory before pregnancy. In addition, postpartum memory Tregs may be exposed to paternal antigens following exposure to seminal fluid, which may benefit the maintenance of memory Tregs. This conjecture remains to be confirmed.

### Fetal cells and fetal microchimerism

The embryo carries antigens that differ from those of the mother. Numerous fetal cells (and fragments) flow into the maternal circulation during normal pregnancy 77, providing abundant fetal and placental antigens for the maternal immune system. Reportedly, the presence of fetal cells in postpartum maternal circulation 78, 79 may cause antigenic activation of memory T cell populations 80, 81. This may lead to the persistence of postpartum immune memory cells. After mating in mice 12-21 days, proliferation fetal cells were unequivocally demonstrated in maternal spleen and bone marrow 82. Bianchi et al. 80 revealed that male fetal progenitor cells could survive in maternal blood for up to 27 years after delivery. Fetal cells and acellular substances are transferred to the maternal circulation during early pregnancy, and fetal cells may survive in the maternal circulation and tissues for life. This substance is called "fetal microchimerism" 83-86.

Recent findings suggested that these microchimeric cells expressing antigenic traits were purposefully retained within mothers and their offspring to promote genetic fitness by improving the outcome of future pregnancies 87. Microchimerism may persist in the maternal circulation post-pregnancy. Reportedly, the rapid increase in Tregs in uterine dLN begins 3 or 4 days after embryo implantation. Embryo implantation triggers early recruitment of amTregs in mouse uterine dLN 10. The proliferation of memory Tregs in early pregnancy is driven by fetal antigens that exhibit Treg specificity. Subsequent studies assessing surface markers of Tregs in dLN failed to detect CD103, CTLA-4, inducible co-stimulator (ICOS), programmed cell death protein-1 (PD-1), CD25, and glucocorticoid-induced changes in tumor necrosis factor receptor-related proteins 10.

### Cytokines

It has been reported that IL-2 can induce the production of memory Tregs 88. Moreover, Low-dose IL-2 induced Tregs expansion can improve recurrent spontaneous abortion in a model of mouse miscarriage. In contrast, a recent study found that IL-2 was non-essential to maintaining CD44^hi^CD62L^low^CCR7^low^ Tregs 89. The role of IL-2 in pregnant memory Tregs warrants further investigations.

The total frequency of memory marker CD27 positive B cells increased during pregnancy, persisted during pregnancy, produced IL-10, and clustered with Foxp3 post cells 7. IL-10 secreted by B cells contributes to the induction and maintenance of placental Tregs and plays a pivotal role in healthy pregnancies. Notably, memory Tregs are primarily maintained by IL-7 in the skin 88, 90. However, the effects of IL-10 and IL-7 on memory Tregs during pregnancy have not been reported.

**Figure 1:**
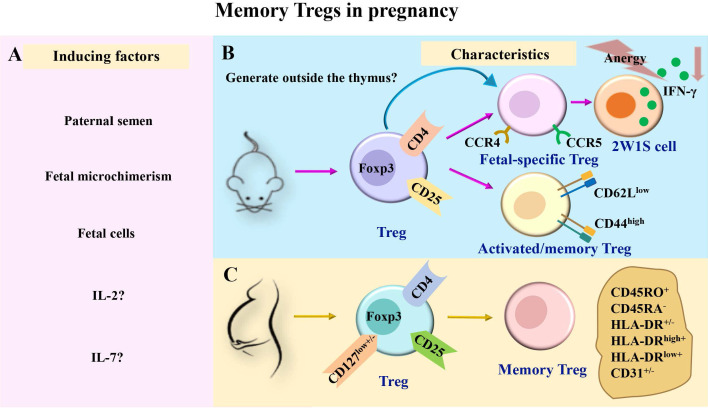
** The induction and characteristics of memory Tregs in pregnancy.** (A) During pregnancy, Tregs in maternal peripheral blood may be induced to generate memory Tregs by paternal semen, fetal microchimerism, fetal cells, and cytokines such as IL-2 and IL-7. (B-C) Previous studies have divided memory Tregs into different subsets in mice and humans. In pregnant mice, memory Tregs were divided into fetal-specific Tregs and activated/memory Tregs (CD4^+^Foxp3^+^CD44^high^CD62L^low^). Among them, fetal-specific Tregs may be generated outside the thymus, and they secreted IFN-γ reduced and exhibited a state of anergy (B). In pregnant women, memory Tregs were divided into HLA-DR^+^ memory Tregs (CD45RA^-^HLA-DR^+^ Tregs), HLA-DR^-^ memory Tregs (CD45RA^-^HLA-DR^-^ Tregs), HLA-DR^low+^ memory Tregs (CD45RA^-^HLA-DR^low+^ Tregs), HLA-DR^high+^ memory Tregs (CD45RA^-^HLA-DR^high+^ Tregs), CD31^+^ memory Tregs (CD45RA^-^CD31^+^ Tregs) and CD31^-^ memory Tregs (CD45RA^-^CD31^-^ Tregs) (C). IL, interleukin; TGF-β, transforming growth factor-β; Tregs, regulatory T cells; Foxp3, forkhead box P3; CCR, chemokine receptor; IFN-γ, interferon-γ.

The maintenance of memory Tregs during pregnancy remains poorly understood. The underlying rationale could be related to the prolonged existence of fetal cells in maternal circulation. It is similar to antigen stimulation at the later stages of persistent infection. Notably, the incidence of PE in the second pregnancy was lower than that in the first pregnancy 3. However, when the interval between two pregnancies is prolonged, the risk of PE continues to increase in women who have repeated pregnancies. Functional changes and maintenance mechanisms of activated memory Tregs need to be comprehensively investigated in future investigations. The induction and characteristics of memory Tregs during pregnancy are shown in Fig. 1.

## Memory Tregs in the second pregnancy

Accumulated evidence indicates that the incidence of pregnancy-related complications such as PE in multiparas were lower than that in nulliparas 3. The memory characteristics of immune cells play a key role in repeated pregnancies. A previous study revealed a population of pregnancy-trained decidual NK cells (PTdNKs) was amplified in the decidua of women with repeated pregnancy when compared with that in the decidua of women during the first pregnancy. PTdNKs were characterized by high expression of NKG2C and leukocyte immunoglobulin-like receptor B1 transcripts. In addition, these cells secrete high levels of vascular endothelial growth factor-α (VEGF-α), which may be beneficial for angiogenesis and pregnancy 13.

Using their established adoptive transfer mouse model, Barton et al. 91 identified fetal-specific T cells. They found that these cells did not increase after the second mating pregnancy with the same partner. However, fetal-specific T cells in the second pregnancy highly expressed CD43 and PD-1, with a lower expression of CD127, which may contribute to the success of subsequent pregnancies to a certain extent 91. The same phenomenon was observed in mouse CD8+T cells in another study by Kinder and colleagues 1. Compared with the first allogeneic pregnancy, the levels of PD-1 and LAG-3, expressed by maternal CD8^+^ T cells stimulated by the fetus, gradually increased during the second pregnancy. Simultaneously, the memory of fetal expressed antigen during the second pregnancy weakened the cytolysis function of maternal CD8^+^ T cells initiated by the previous pregnancy, thus affording protection against fetal loss 1.

Unlike CD8^+^T cells, which weakened the cytolysis function in the second pregnancy, fetal-specific Tregs maintained at a high level after parturition in mice and expanded rapidly in the second pregnancy, showing the characteristics of immune tolerance. But they are conducive to the maintenance of pregnancy and against fetal loss. Rowe et al. 17 revealed that during the second pregnancy, the accelerated expansion of Tregs was almost entirely driven by proliferating fetal-specific Foxp3^+^ cells retained from the previous pregnancy, which was further conducive to pregnancy. The amplification rate was considerably faster in fetal-specific Foxp3^+^ cells than in nTregs. They also found an interesting phenomenon that 2W1S^+^ CD4 fetal-specific T cells maintained reduced interferon-γ (IFN-γ) secretion after pregnancy, that is, they showed anergy. And the anergy between maternal CD4 cells was not inherent in cells but maintained by the postpartum environment 17. Another study found that compared with the first pregnancy, neonatal mortality was higher when Tregs were exhausted during the third trimester of the second pregnancy. The mortality rate associated with complete depletion was higher than that observed following partial depletion 39. In addition, during repeated pregnancy, the newborns who survived in the group with partial or complete lack of Tregs were thinner and presented worse outcomes than their control group counterparts. These results support the concept that pregnancy imprints protective regulatory memories 39. Furthermore, the amplification of Tregs during the second pregnancy was related to maternal-fetal tolerance, as well as neonatal health.

Few studies have been documented in the function of memory Tregs during the second pregnancy in humans. These studies focused on the expansion of immune memory cells in the second pregnancy to produce immune tolerance, which is further conducive to the maintenance of pregnancy. Clarifying the function and changes of memory Tregs during the second pregnancy is pivotal to reducing pregnancy-related complications during repeated pregnancies and elucidating the disease mechanism.

## Memory Tregs in pathological pregnancy

An imbalance in immune tolerance can induce pregnancy complications. A study assessing 763,795 patients has reported that the risk of PE was 4.1% during the first pregnancy and 1.7% during subsequent pregnancies. However, a PE risk of 14.7% was observed during the second pregnancy in women who had experienced PE during the first pregnancy and was approximately 1% in multiparous women without a history of PE 92. Moreover, a large meta-analysis of data from more than 1.5 million patients found that women who experienced stillbirth, preterm birth, and fetal growth restriction before pregnancy exhibited a significantly increased risk for each condition during subsequent pregnancies 93. These results indicate that good pregnancy memory may lead to a good pregnancy outcome, while poor pregnancy memory may be associated with the subsequent increase in the incidence of pregnancy-complicated diseases.

Recently, changes in memory Tregs have been detected in association with pregnancy complications, which could be employed as a new target for diagnosing and treating pregnancy complications. Previous studies assessed changes in memory Tregs during pregnancy complications in PE, gestational diabetes mellitus (GDM), preterm delivery, and the success of assisted reproductive technology (ART).

### PE

PE affects 5%-8% of pregnancies and is the main cause of fetal and maternal mortality and morbidity 94. In women with PE, there is a negative correlation between Tregs and memory B cells, which is characterized by a systematic decrease of Tregs and an increase of memory B cells 9. But in memory Tregs, Clonal expansion of the CD4^+^CD25^HI^CD127^-^CD45RA^-^ Treg population has been observed in the decidua of healthy term pregnancies. The failure of clonal expansion may be related to the occurrence of PE 95.

In patients with PE, RTE-Tregs differentiate into CD31^+^ memory Tregs rather than CD31^-^ memory Tregs. CD31^+^ memory Tregs reportedly decrease during normal pregnancy 60. However, the underlying mechanism remains unclear. A study by Steinborn et al. 68 revealed that the percentages of DR^low+^ CD45RA^-^ Tregs and DR^high+^CD45RA^-^ Tregs were significantly increased in pregnant women with PE. Moreover, the percentage of DR^high+^CD45RA^-^ Tregs was significantly upregulated in patients with hemolysis, elevated liver enzymes, and low platelets (HELLP) syndrome. Nevertheless, this study did not define DR^low+^CD45RA^-^ Tregs and DR^high+^CD45RA^-^ Tregs as memory Tregs, although CD45RA^-^ is typically defined as a memory marker.

### GDM

GDM refers to diabetes mellitus with normal glucose metabolism or potentially impaired glucose tolerance before pregnancy, which appears or is diagnosed during pregnancy. The reported incidence among pregnant women ranges between 2%-9% 96 and appears to be growing. Recent studies have revealed that GDM is characterized by chronic systemic inflammation and increased humoral immune responses 97. Tregs can reportedly modulate the excessive enhancement of immune responses. It has been shown that although the inhibitory activity of Tregs was significantly decreased in patients with GDM, no difference in the total percentage of Tregs was detected.

Interestingly, in the subgroup analysis of Tregs, a considerably higher number of DR-memory Tregs was observed in dietary-adjusted pregnant patients with GDM than in healthy pregnancies. However, insulin-dependent patients with GDM exhibited considerably higher levels of DR^low+^ Tregs and DR^high+^ Tregs 59. It has been suggested that the cellular function of pregnancy memory may be related not only to immunity but also to metabolism. Indeed, previous studies have suggested that proliferation depends on glycolysis, and memory depends on fatty acid oxidation 98. However, whether the increase in memory Tregs is related to metabolism remains to be determined.

Taken together, these studies suggest that DR^low+^ memory Tregs and DR^high+^ memory Tregs may be unfavorable for pregnancy. This finding is similar to the increase in DR^low+^ memory Tregs and DR^high+^ memory Tregs observed in PE. However, in normal pregnancy, these two cell types are decreased 68. DR^+^ Tregs and DR^-^ Tregs increased in the early stage of normal pregnancy, as shown in Table 2. However, the mechanism is not clear, especially in the second pregnancy.

### Premature birth

In developed countries, premature birth remains the primary cause of morbidity and mortality, with a reported incidence rate of approximately 5%-9% 99. Gomez-Lopez et al. 39 reported the immunomodulatory effects of Tregs during the third stage of pregnancy in mice. Treg deficiency may lead to idiopathic preterm births and poor perinatal outcomes. Interestingly, the authors revealed that complete depletion of Tregs during the third trimester of the second pregnancy minimally impacted the preterm birth rate while significantly influencing neonatal survival 39. Another study reported that the percentages of DR^-^CD45RA^-^ Tregs and DR^low+^CD45RA^-^ Tregs were significantly higher in human preterm births than in healthy pregnancies. However, no change in Treg subsets was detected in patients with simple cervical insufficiency and no preterm birth 68. Accordingly, it can be suggested that distinct mechanisms possibly underlie preterm birth and cervical insufficiency.

### ART outcome

Memory Tregs may be associated with ART outcome. In the study of Schlossberger et al. 18, they determined the association of total Treg pool and distinct Treg subsets (na ¨ıve CD45RA^+^ Tregs, HLA-DR^-^ and HLA-DR^+^ memory Tregs) with the success of IVF/ICSI treatment. They found that the percentage of Tregs within the total CD4^+^ T cell pool was not different between successfully and non-successfully IVF/ICSI-treated women. However, there were a decreased percentage of na ¨ıve CD45RA^+^ Tregs and an increased percentage of HLA-DR^-^ memory Tregs within the total Treg pool in non-successfully IVF/ICSI-treated women. They suggested that the ART success might be related to composition of the total Treg pool with na ¨ıve CD45RA^+^ Tregs and HLA-DR^-^ memory Tregs.

### RPL and miscarriage with normal fetal karyotype

Extensive studies have shown that systemic and local maldistribution and dysfunction of Tregs could be one of the etiologies of RPL and miscarriage with normal fetal karyotype 95, 100. However, there are less studies targeting memory Tregs in RPL and miscarriage, which highlights this topic as future research direction. In a recent study, Tsuda et al. 95 found that the frequency of CD4^+^CD45RA^-^CD25^+^CD127^low/-^ effector Tregs (also defined as one of memory Treg subsets) among CD4^+^CD25^+^CD127^low/-^ total Tregs in decidua were significantly lower in miscarriage with normal chromosomal karyotyped embryo than 1^st^ trimester normal pregnancy. They suggested that the decreased number of decidual effector Tregs might be related to the pathogenesis of miscarriage with normal fetal karyotype, for the effector Treg subset might contain fetal antigen specific populations in humans.

As is known that the number of miscarriages increases, the probability of miscarriage in the next pregnancy also increases. However, whether memory Tregs are correlated with the occurrence of repeated miscarriages with normal fetal karyotype is unknown and needs further investigations.

Table 2 summarizes the changes of memory Tregs in healthy and pathological pregnancies in humans. It seems that there are higher different memory Treg subsets in different pregnancy complications. However, the exact role of the different memory Treg subsets in the subsequent pregnancies of women who experienced pregnancy-related complications during their first pregnancy remains unclear and needs further investigations.

## Conclusions

In conclusion, paternal semen, fetal cells, fetal microchimerism, and cytokines may induce the transformation of Tregs into memory Tregs during pregnancy. They include fetal-specific Tregs and Tregs expressing memory makers. During normal pregnancy, the good memory Tregs contribute to embryo implantation and play a role in immune tolerance. The increase in detrimental memory Tregs may be related to pregnancy complications. In human pregnancy, data regarding the changes and functions of memory Tregs in repeated pregnancies are limited. In mice, the role of memory Tregs in pathological pregnancy is lacking. An in-depth study on the role of memory Tregs in normal and pathological pregnancies, especially in the subsequent pregnancy, will help to uncover the mechanism underlying pregnancy-related diseases, which could further afford novel strategies for the clinical diagnosis and treatment of pathological pregnancies. Moreover, identifying more conclusive markers for memory Tregs' function is extremely important to fully elucidate the role of memory Tregs in human pregnancy.

## Figures and Tables

**Table 1:** Summary of the studies on memory Tregs in pregnancy

Grouping	Markers of memory Tregs	Sampling times and sources for memory Tregs	Proportion of memory Tregs in normal pregnancy	Main conclusions	References
♀B6 ×♂Balb/c-2W1S; ♀B6 ×♂B6-2W1S; ♀B6 ×♂Balb/c.	CD4^+^2W1S^+^ Foxp3^+^	Virgin; E 11.5; E 18.5; PP 2; PP 14; PP 30; PP 100 (in spleen and lymph nodes)	(Percentage of Foxp3^+^ among 2W1S^+^CD4^+^ cells) Virgin: 7%; E11.5: 21.1%; E18.5: 45.1%; PP2: 60.1%; PP14: 20.1%; PP30: 19.6%; PP100: 18.4%.	Pregnancy imprints Foxp3^+^CD4 cells to maintain the protective regulatory memory to the fetal antigen.	17
♀Balb/c ×♂B6	amTregs: CD4^+^Foxp3^+^CD44^high^CD62L^low^	dpi 1; dpi 4; dpi 6; dpi 7; dpi 10; dpi 12 (in dLN and ndLN)	In dLN, the frequency of amTregs from dpi 6 to dpi 10 increased.	Early recruitment of amTregs in uterine dLNs was triggered by embryo implantation.	10
♀Foxp3^DTR^ ×♂BALB/C; Tregs depletion with DT.	Tregs: CD45^+^CD3^+^CD4^+^CD25^+^Foxp3^+^	Delivery; 1w, 2w, and 3 w of postpartum (in decidua, myometrium, peripheral blood, placenta)	None.	The enhanced Tregs expansion during the second pregnancy was related to maternal-fetal tolerance, as well as the health of the newborn.	39
Patients undergoing IVF / ICSI: Pregnancy group (n = 36); Nonpregnant group (n = 160).	DR^+^ Tregs: D45RA^-^HLA-DR^+^ Tregs; DR^-^ Tregs: CD45RA^-^HLA-DR^-^ Tregs	1 hour before embryo transfer; 7w; 14w; 21w; 28w (in peripheral blood)	1 hour before embryo transfer in pregnancy group (Percentage in total Tregs); DR^+^ Tregs: 27.0%; DR^-^ Tregs: 30.1%.	Compared with that in women with successful pregnancy, the percentage of DR^-^Tregs was increased in non-pregnant women.	18
Healthy pregnant women (n = 64); Dietary-adjusted GDM ( n = 21); Insulin-dependent GDM (n = 40).	DR^-^ Tregs: CD45RA^-^HLA-DR^-^Tregs; DR^low+^ Tregs: CD45RA^-^HLA-DR^low+^ Tregs; DR^high+^ Tregs: CD45RA^-^HLA-DR^high+^Tregs.	24 - 41 w (in peripheral blood)	Healthy pregnant group (Percentage in total Tregs): DR^+^ Treg: 22.8%; DR^low+^ Treg: 20.3%; DR^high+^ Treg: 2.5%; DR^-^Treg: 29.9% .	① The percentage of DR^-^Tregs was significantly higher in patients with dietary-adjusted GDM than that in healthy pregnancies; ② The percentages of DR^low+^ Tregs and DR^high+^ Tregs were significantly higher in patients with insulin-dependent GDM than those in healthy pregnancies.	59
Non-pregnant women (n=31); Healthy pregnant women (n=169); PE ( n = 37).	CD31^+^ memory Tregs: CD45RA^-^CD31^+^ Tregs; CD31^-^ memory Tregs: CD45RA^-^CD31^-^ Tregs (Tregs: CD4^+^CD127^low+/-^Foxp3^+^).	1^st^ trimester; 2^nd^ trimester; 3^rd^ trimester; term (in peripheral blood)	3^rd^ trimester (Percentage in total Tregs): CD31^+^memory Tregs: 4%; CD31^-^memory Tregs: 66%.	① At the beginning of pregnancy, RTE-Tregs differentiated into CD31^-^memory Tregs and were maintained until term delivery. ② In PE group, CD45RA^-^CD31^+^ memory Tregs were significantly increased.	60
Non-pregnant fertile women (n = 31); Healthy pregnant women (n = 135) ; PE (n = 27); HELLP (n = 15) ; CI (n = 30); PL (n = 24).	CD45RA^-^HLA-DR^-^ Tregs; CD45RA^-^HLA-DR^low+^ Tregs; CD45RA^-^HLA-DR^high+^ Tregs.	24-42 weeks' gestation (in peripheral blood)	Healthy pregnant group (Percentage in Tregs): CD45RA^-^HLA-DR^-^Treg: 30.4%; CD45RA^-^HLA-DR^low+^Treg: 23.6%; CD45RA^-^HLA-DR^high+^Treg: 4.8%.	PE and PL were characterized by distinct Treg subsets accompanied by a significant decrease in their suppressive activity.	68

*Note:* Tregs not specifically defined in the table are CD4^+^CD127^low+/-^CD25^+^Foxp3^+^. amTregs: activated/memory Tregs; CI: cervical insufficiency; dLN: draining lymph node; dpi: day postimplantation; DT: diphtheria toxin; DTR: diphtheria toxin receptor; DR^+^ Tregs: HLA-DR^+^ memory Tregs; DR^-^ Tregs: HLA-DR^-^ memory Tregs; DR^high+^ Tregs: HLA-DR^high+^ memory Tregs; DR^low+^ Tregs: HLA-DR^low+^ memory Tregs; E: embryonic day; Foxp3^+^: forkhead box P3^+^; GDM: gestational diabetes mellitus; HELLP: haemolysis-elevated liver enzyme levels-low platelet count syndrome; IVF/ICSI: *in vitro* fertilization/intracytoplasmic sperm injection; ndLN: non-dLN; PE: preeclampsia; PL: preterm labour necessitating preterm delivery; PP: post-partum day; RTE-Tregs: recent thymic emigrant-regulatory T-cells; Tregs: Regulatory T cells; w: weeks of gestation; 1^st^ trimester: the first trimester; 2^nd^ trimester: the second trimester; 3^rd^ trimester: the third trimester.

**Table 2:** Changes of memory Tregs in healthy and pathological pregnancies

Memory Tregs	Mouse or human	During normal pregnancy			PE	Dietary GDM	Insulin GDM	HELLP	PL	Failure of ART	References
		1^st^ trimester	2^nd^ trimester	3^rd^ trimester							
amTregs	mouse	↑									10
fetal-specific Tregs	mouse	↑	↑	↑							17
DR^+^ Tregs	human	↑	↓	↓							18
DR^-^ Tregs	human	↑				↑			↑	↑	18 68
DR^low+^ Tregs	human		↓		↑		↑		↑		59 68
DR^high+^ Tregs	human		↓		↑		↑	↑			59 68
CD31^+^ memory Tregs	human	↓			↑						60
CD31^-^ memory Tregs	human	↑									60

*Note:* amTregs: activated/memory Tregs (CD4^+^Foxp3^+^CD44^high^CD62L^low^); ART: Assisted Reproductive Technology; CD31^+^ memory Tregs: (CD45RA^-^CD31^+^ Tregs); CD31^-^ memory Tregs: (CD45RA^-^CD31^-^ Tregs); DR^+^ Tregs: HLA-DR^+^ memory Tregs (CD45RA^-^HLA-DR^+^ Tregs); DR^-^ Tregs: HLA-DR^-^ memory Tregs (CD45RA^-^HLA-DR^-^ Tregs); DR^low+^ Tregs: HLA-DR^low+^ memory Tregs (CD45RA^-^HLA-DR^low+^ Tregs); DR^high+^ Tregs: HLA-DR^high+^ memory Tregs (CD45RA^-^HLA-DR^high+^ Tregs); GDM: gestational diabetes mellitus; HELLP: haemolysis-elevated liver enzyme levels-low platelet count syndrome; PE: preeclampsia; PL: irresistible preterm delivery; Tregs: Regulatory T cells; 1^st^ trimester: the first trimester; 2^nd^ trimester: the second trimester; 3^rd^ trimester: the third trimester.

## Abbreviations

amTregs: activated/memory Tregs
ART: assisted reproductive technology
CCR: chemokine receptor
dLN: draining lymph node
dpc: days postcoital
dpi: day postimplantation
DT: diphtheria toxin
DTR: diphtheria toxin receptor
Foxp3^+^: forkhead box P3^+^
GDM: gestational diabetes mellitus
HELLP syndrome: patients with hemolysis, elevated liver enzymes, and low platelets syndrome
HLA: human leukocyte antigen
ICOS: inducible co-stimulator
IFN-γ: interferon-γ
IL: interleukin
IVF/ICSI: *in vitro* fertilization/intracytoplasmic sperm injection
MHC: major histocompatibility complex
NK: natural killer
Ova: Ovalbumin
PD-1: Programmed Cell Death Protein-1
PE: preeclampsia
PTdNKs: pregnancy-trained decidual NK cells
pTregs: Peripheral-derived Tregs
RPL: recurrent pregnancy loss
T_CM_: central memory T cells
Teffs: effector T cells
T_EM_: effector memory T cells
TGF-β: transforming growth factor-β
Tregs: Regulatory T cells
T_RM_: tissue-resident memory T cells
VEGF-α: vascular endothelial growth factor-α
